# Exosomes released by hepatocarcinoma cells endow adipocytes with tumor-promoting properties

**DOI:** 10.1186/s13045-018-0625-1

**Published:** 2018-06-14

**Authors:** Shihua Wang, Meiqian Xu, Xiaoxia Li, Xiaodong Su, Xian Xiao, Armand Keating, Robert Chunhua Zhao

**Affiliations:** 1 Center of Excellence in Tissue Engineering, Institute of Basic Medical Sciences, Peking Union Medical College Hospital, Chinese Academy of Medical Sciences, School of Basic Medicine Peking Union Medical College, Beijing, 100005 China; 2 Department of Genetics and Cell Biology, Basic Medical College, Qingdao University, 308 Ningxia Road, Qingdao, 266071 China; 3 Cell Therapy Translational Research Laboratory, Princess Margaret Cancer Centre, Toronto, Ontario M5G 2M9 Canada; 4 Institute of Biomaterials and Biomedical Engineering, University of Toronto, Toronto, Ontario M5G 2M9 Canada; 5 Institute of Medical Science, University of Toronto, Toronto, Ontario M5G 2M9 Canada

**Keywords:** Exosomes, Adipocyte, HCC, MSCs, NF-κB

## Abstract

**Background:**

The initiation and progression of hepatocellular carcinoma (HCC) are largely dependent on its local microenvironment. Adipocytes are an important component of hepatic microenvironment in nonalcoholic fatty liver disease (NAFLD), which is a significant risk factor for HCC. Given the global prevalence of NAFLD, a better understanding of the interplay between HCC cells and adipocytes is urgently needed. Exosomes, released by malignant cells, represent a novel way of cell-cell interaction and have been shown to play an important role in cancer cell communication with their microenvironment. Here, we explore the role of HCC-derived exosomes in the cellular and molecular conversion of adipocytes into tumor-promoting cells.

**Methods:**

Exosomes were isolated from HCC cell line HepG2 and added to adipocytes. Transcriptomic alterations of exosome-stimulated adipocytes were analyzed using gene expression profiling, and secretion of inflammation-associated cytokines was detected by RT-PCR and ELISA. In vivo mouse xenograft model was used to evaluate the growth-promoting and angiogenesis-enhancing effects of exosome-treated adipocytes. Protein content of tumor exosomes was analyzed by mass spectrometry. Activated phospho-kinases involved in exosome-treated adipocytes were detected by phospho-kinase antibody array and Western blot.

**Results:**

Our results demonstrated that HCC cell HepG2-derived exosomes could be actively internalized by adipocytes and caused significant transcriptomic alterations and in particular induced an inflammatory phenotype in adipocytes. The tumor exosome-treated adipocytes, named exo-adipocytes, promoted tumor growth, enhanced angiogenesis, and recruited more macrophages in mouse xenograft model. In vitro, conditioned medium from exo-adipocytes promoted HepG2 cell migration and increased tube formation of human umbilical vein endothelial cells (HUVECs). Mechanistically, we found HepG2 exosomes activated several phopho-kinases and NF-κB signaling pathway in exo-adipocytes. Additionally, a total of 1428 proteins were identified in HepG2 exosomes by mass spectrometry.

**Conclusions:**

Our results provide new insights into the concept that tumor cell-derived exosomes can educate surrounding adipocytes to create a favorable microenvironment for tumor progression.

## Background

Hepatocellular carcinoma (HCC) now represents the fifth most common cancer worldwide and the third leading cause of cancer-related mortality [1, 2]. Although both diagnostic and therapeutic strategies for HCC have improved over the past decades, the 5-year survival rate only is around 10%, and HCC continues to be a global health issue, especially in Asian countries [3, 4]. Emerging evidence suggested that nonalcoholic fatty liver disease (NAFLD), a common disorder in obese people, is a significant risk factor for HCC [5, 6]. Given the global prevalence of obesity, there is the looming threat of a rapidly rising occurrence of NAFLD-related HCC. Therefore, it is urgent and paramount to understand the mechanisms by which NAFLD contributes to HCC development.

Tumor behavior is determined by not only the malignant potential of tumor cell itself but also the signals from its microenvironment. Thus, it is clear that the crosstalk between tumor cells and their surrounding microenvironment is crucial for HCC development. In NAFLD, the hepatic microenvironment comprises multiple cell lineages including endothelial cells, hepatic satellite cells, immune cells, and adipocytes [7, 8]. Previous studies have focused intensively on the interactions between HCC cells and a wide variety of immune cells such as Kupffer cells, NK cells, T cells, and several antigen-presenting cells. For example, necrotic debris of HCC cells can induce potent IL-1β release by macrophages which subsequently promote HCC metastasis in mouse models [9]. The work done by Wolf et al. showed that hepatic NKT cells promoted NAFLD by secreting LIGHT and activated NF-κB signaling in hepatocytes to enhance malignant transformation [10]. However, the interplay between the HCC cells and adjacent adipocytes remains poorly understood so far.

Currently, how cancer cells communicate with their local and distant microenvironment is undergoing a re-evaluation with the discovery of a novel way of cell-cell interaction exosomes [11, 12]. In addition to diffusible factors, such as growth factors, cytokines, and extracellular bioactive molecules, exosomes are small membrane vesicles that are released by many different cell types, including cancer cells. Increasing evidence suggests that tumor-derived exosomes support tumor development and progression by generating a favorable milieu through immune suppression, angiogenesis enhancement, extracellular matrix remodeling, and stromal cell conversion [13–15]. Exosome-mediated transfer of proteins, DNA, noncoding RNAs, and mRNAs could induce phenotypic changes in target cells [16]. In melanoma, the tumor-derived exosomes educated bone marrow progenitors toward a pro-metastatic phenotype through the receptor tyrosine kinase MET [17]. In pancreatic cancer, the secreted exosomes induced lipolysis in subcutaneous adipose tissue [18]. In HCC, exosomes derived from metastatic HCC cell lines significantly enhanced the migratory and invasive abilities of nonmotile hepatocytes [19]. However, to our knowledge, no study has reported on the effects of tumor-derived exosomes on adipocytes, which represent an abundant cell type within tumor microenvironment in overweight patients.

In this study, we explored the role of HCC-derived exosomes in the cellular and molecular conversion of adipocytes into tumor-promoting cells. Our results demonstrated that HCC cell line HepG2-derived exosomes could be actively internalized by adipocytes differentiated from mesenchymal stem cells (MSCs) and caused significant transcriptomic alterations, and in particular, induced an inflammatory phenotype in adipocytes. The tumor exosome-treated adipocytes, named exo-adipocytes, promoted tumor growth, enhanced angiogenesis, and recruited more macrophages in mouse xenograft model. In vitro, conditioned medium from exo-adipocytes promoted HepG2 cell migration and increased tube formation of human umbilical vein endothelial cells (HUVECs). Mechanistically, we found HepG2 exosomes activated several kinases and NF-κB signaling pathway in exo-adipocytes. Our findings showed for the first time that HCC-derived exosomes could convert adipocytes into tumor-promoting cells, which may provide new insights into understanding the interactions between tumor cells and surrounding microenvironment.

## Methods

### Cell culture

Human adipose tissues and umbilical cords were obtained according to the procedures approved by the Ethics Committee at the Chinese Academy of Medical Sciences and Peking Union Medical College. MSCs were isolated and culture-expanded from healthy volunteers as previously reported [20]. Passage 3 MSCs were used for following experiments. To obtain adipocytes, MSCs were induced under adipogenic differentiation medium, which is high glucose of Dulbecco’s modified Eagle’s medium (H-DMEM) supplemented with 10% FBS, 1 μM dexamethasone, 0.5 mM 3-isobutyl-1-methylxanthine, and 5 μg/mL 0.1 mM l-ascorbic acid. Adipocytes were characterized by Oil Red O staining according to the manufacture’s (Beyotime Biotechnology) instructions. HUVECs were isolated and cultured as routinely described [21]. HCC cell line HepG2 was purchased from cell bank at the Chinese Academy of Medical Sciences and cultured in DF12 containing 10% FBS, penicillin (100 U/mL), and streptomycin (100lg/mL) at 37 °C with 5% CO_2_.

### Exosomes isolation

Exosome extraction was performed as previously described [22]. Briefly, HepG2 cells were cultured in serum-free DF12 medium for 24 h. Then, the culture medium was collected and centrifuged at 800*g* for 5 min and additional 2000*g* for 10 min to remove lifted cells. The supernatant was subjected to filtration on a 0.1-mm-pore polyethersulfone membrane filter (Corning) to remove cell debris and large vesicles, followed by concentration by a 100,000-Mw cutoff membrane (CentriPlus-70, Millipore). The volume of supernatant was reduced from approximately 250–500 mL to less than 5 mL. The supernatant was then ultracentrifuged at 100,000*g* for 1 h at 4 °C using 70Ti Rotor (Beckman Coulter). The resulting pellets were resuspended in 6 mL PBS and ultracentrifuged at 100,000*g* for 1 h at 4 °C using 100Ti Rotor (Beckman Coulter). In the experiments involving HepG2 exosomes, we use PBS as a negative control.

### Transmission electron microscopy

Purified exosomes were fixed with 1% glutaraldehyde in PBS (pH 7.4). After rinsing, a 20-uL drop of the suspension was loaded onto a formvar/carbon-coated grid, negatively stained with 3% (*w*/*v*) aqueous phosphotungstic acid for 1 min, and observed by transmission electron microscope.

### Quantitative real-time polymerase chain reaction

Total RNA was extracted using TRIzol (Invitrogen) according to the manufacturer’s instruction, and cDNA was prepared. Real-time PCR amplification was performed in triplicates according to the procedures reported previously [23]. Relative expression of mRNA was evaluated by the 2-ΔΔCt method and normalized to the expression of GAPDH.

### Western blotting

Proteins were extracted with radioimmunoprecipitation (RIPA) lysis buffer with PMSF, quantified by BCA Protein Assay Kit (Beyotime). Western blot was performed in triplicates according to the procedures reported previously [24]. GAPDH was used as an internal control. We used the following antibodies: p-AKT (1:2000; rabbit IgG, CST, 4060T), p-ERK1/2 (1:5000; rabbit IgG, Abcam, ab76299), p-STAT5α (1:1000; rabbit IgG, Abcam, ab30648), p-GSK (1:5000; rabbit IgG, Abcam, ab75814), AKT (1:1000; mouse IgG, proteintech, 60203-2-Ig), ERK1/2 (1:1000; rabbit IgG, proteintech, 16443-1-AP), STAT5α (1:1000; rabbit IgG, Abcam, ab32043), GSK3β (1:1000, rabbit IgG, proteintech, 22104-1-AP), CD63 (1:500; rabbit IgG, proteintech, 25682-1-AP), TSG101 (1:500; rabbit IgG, Abcam, ab83), HSP70 (1:100; rabbit IgG, SBI, EXOAB-KIT-1), calnexin (1:2000; rabbit IgG, CST, 2433s), GAPDH (1:10000; rabbit IgG, proteintech, 10494-1-AP) (1:10000; mouse IgG, proteintech, 60004-1-Ig), HRP-conjugated anti-rabbit-IgG (NeoBioscience), HRP-conjugated anti-goat-IgG (NeoBioscience), and HRP-conjugated anti-mouse-IgG (NeoBioscience).

### Cytokine analysis

Culture medium was collected 24 h after the treatment with or without exosomes. The concentrations of all cell cytokines in supernatants were measured using ELISA kits (BD Technologies).

### Immunofluorescence staining

The cultured cells were fixed at 4 °C in ice-cold methanol for 10 min, washed three times in phosphate-buffered saline (PBS), and then permeabilized in 0.1% Triton X-100/PBS for 10 min at room temperature. Nonspecific binding was blocked with 0.5% Tween-20/PBS containing 1% bovine serum albumin (BSA) for 30 min. The primary antibodies were incubated at 4 °C overnight. The secondary antibodies were incubated for 1 h at room temperature. The incubated cells were washed in PBS, and Hoechst 33342 (Sigma-Aldrich) was used to visualize nuclei. p65 antibody (10745-1-AP) was purchased from Proteintech.

### Mouse xenograft experiments

Nude mice were purchased from the Laboratory Animal Center of the Chinese Academy of Medical Sciences (Beijing, China). Animal use and experimental procedures were approved by the Animal Care and Use Committee of the Chinese Academy of Medical Sciences. Mice were randomly divided into three groups, one group received a subcutaneous injection of 2 × 10^5^ exo-adipocytes and 2 × 10^6^ HepG2 cells, one group received 2 × 10^5^ adipocytes and 2 × 10^6^ HepG2 cells, and the last one received 2 × 10^6^ HepG2 cells. The tumor weight was measured after 4 weeks. The tumor tissues were fixed with 10% PFA. Each group was treated with HE, IL-6, Ki67, CD31, and F4/80 staining.

### Tube formation assay in Matrigel

In vitro capillary network formation was determined by performing a tube formation assay in Matrigel (BD Biosciences). 1 × 10^4^ HUVECs were plated on a growth factor-reduced Matrigel (BD)-coated 96-well plate in triplicates with 100 uL serum-free medium (control), exo-adipocyte-conditioned medium, or adipocyte-conditioned medium. After 8 h of incubation, tube formation was examined by microscopy (Olympus, Tokyo, Japan), and the branch density and tube length were quantified by randomly selecting three fields per well.

### Statistical analysis

Data are presented as mean ± SD. Comparisons between groups were analyzed via Student’s *t* test. Differences were considered statistically significant at *P < 0.05, **P < 0.01, and ***P < 0.001.

## Results

### HepG2 exosomes are actively internalized by adipocytes

**Fig. 1:**
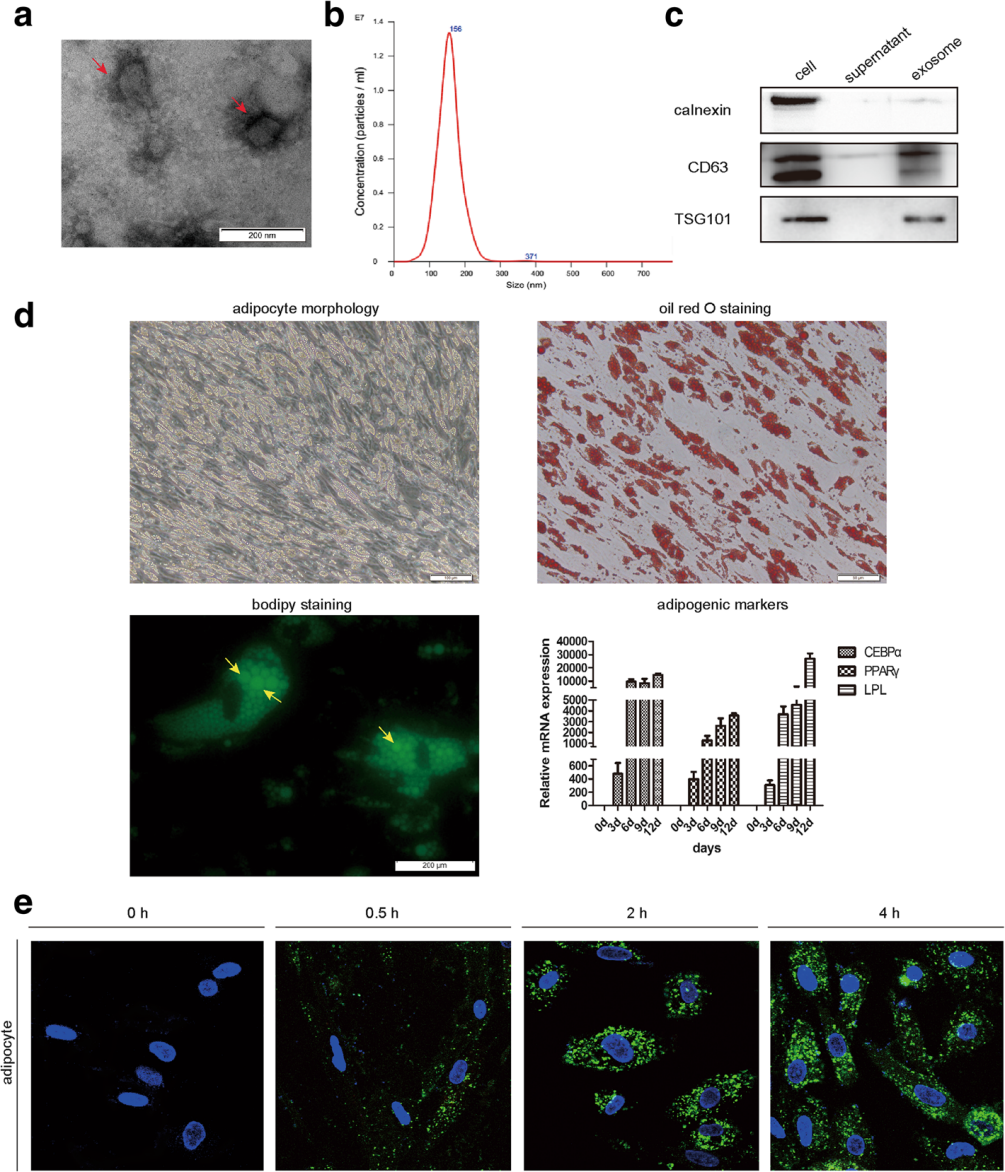
HepG2 cell-secreted exosomes are actively incorporated by adipocytes in culture. **a** A representative electron microscopy image of purified HepG2 exosomes showed morphology. Scale bar, 200 nm. **b** The nanoparticle size distribution for HepG2 exosomes was obtained by NTA. The particle size is between 0 and 300, with a peak around 100 nm. **c** Western blot analysis of exosome marker CD63, TSG101, and cell-specific marker calnexin. Positive control was HepG2 cell lysate, and negative control was culture medium. **d** Characterization of adipocytes differentiated from MSCs by morphology, Oil Red O staining, BODIPY staining, and expression of specific adipogenic marker genes CEBPɑ, PPARɤ, and LPL. **e** Adipocytes were incubated with 70 ng/mL PKH67-labeled HepG2 exosomes for the indicated times, and uptake of exosomes was determined by fluorescence confocal microscopy

To investigate whether exosomes are secreted by hepatocarcinoma cell HepG2 and play a role in their communication with adipocytes, we used differential centrifugation to purify exosomes from the supernatant of HepG2 cells. Isolated particles were observed under transmission electron microscopy and found to present characteristics of exosomes, with typical appearance and diameter ranging from 30 to 200 nm (Fig. 1a). The nanoparticle size distribution for the HepG2 exosomes was further obtained by NTA, and the peaks of particle size were ~ 100 nm, within the expected size of exosomes (Fig. 1b). Enrichment for exosome marker CD63, TSG101, and the absence of the cell-specific marker calnexin was demonstrated by Western blot (Fig. 1c). Adipocytes were differentiated from human mesenchymal stem cells (MSCs) under adipogenic induction conditions. Figure 1d characterizes the MSC-derived adipocytes by morphology, Oil Red O staining, BODIPY staining, and expression of specific adipogenic marker genes during adipogenic differentiation of MSCs. To examine if adipocytes might be targets of HepG2-derived exosomes, a lipid-associating fluorescent dye, PKH67, was used to label exosome preparations and then incubated with adipocytes. Exosome incorporation was observed 0.5 h after treatment, and exosomes accumulated in adipocytes over time (Fig. 1e). Collectively, we show that HepG2 cells secrete exosomes, which are actively incorporated in vitro by adipocytes.

### HepG2 exosomes induce an inflammatory phenotype in adipocytes

**Fig. 2:**
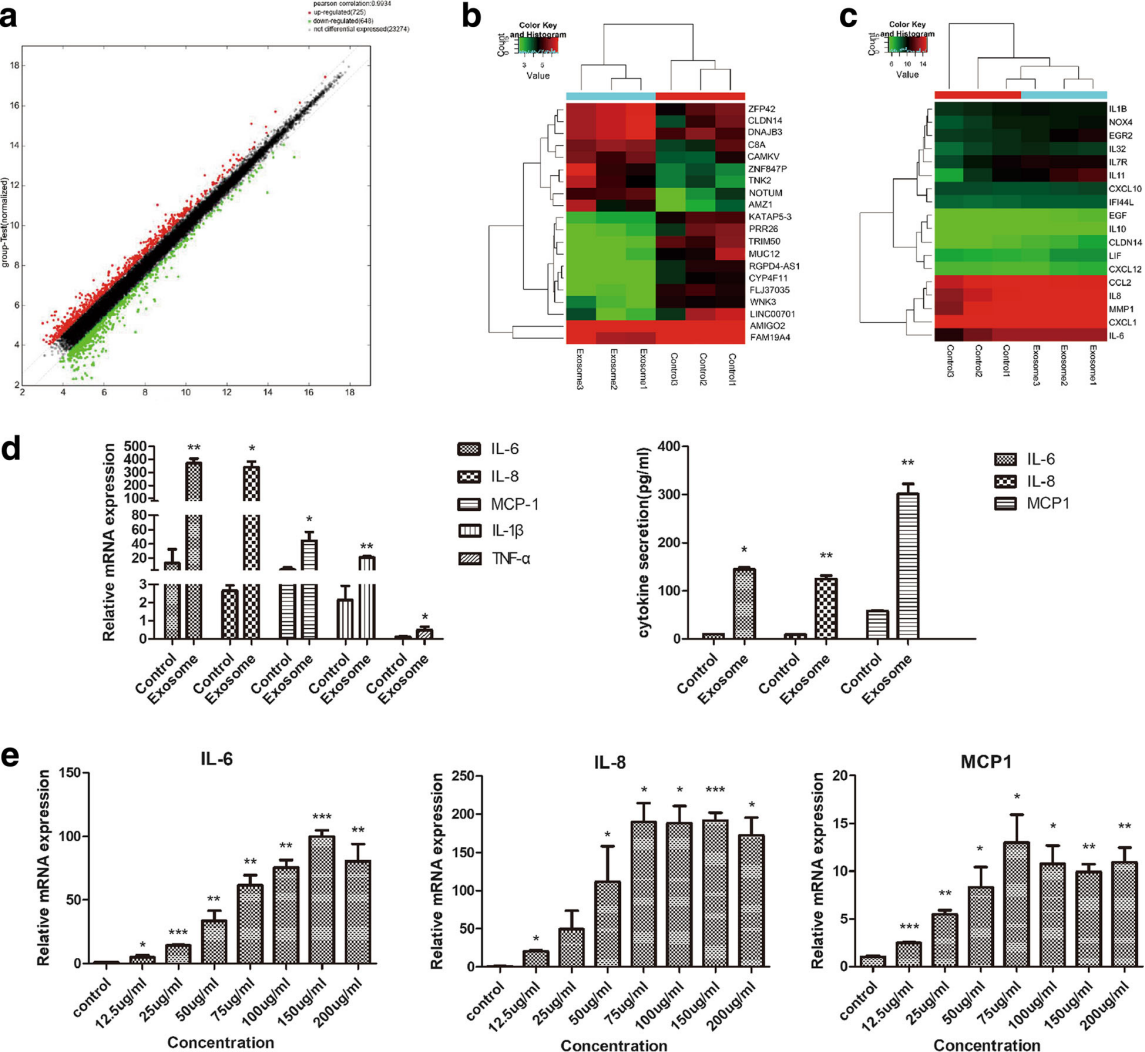
HepG2 exosomes change the transcriptome of adipocytes and induce production of inflammatory cytokines. **a** The scatter plot assessed the gene expression variations between exosome-treated adipocytes and control. The red dots indicate upregulated genes and green dots downregulated genes. **b** The top 10 up- and downregulated genes in HepG2 exosome-treated adipocytes compared to control (E represents exosome-treated adipocytes, C represents control). **c** Unsupervised hierarchical clustering based on the expression of genes associated with inflammation. **d** Enhanced release of IL-6, IL-8, and MCP-1 was detected by qRT-PCR (left) and ELISA (right) (*P < 0.05, **P < 0.01, ***P < 0.001). **e** A partial dose-dependent effect of exosomes on the secretion of IL-6, IL-8, and MCP-1 (*P < 0.05, **P < 0.01, ***P < 0.001)

To determine the effects of HepG2 exosomes on adipocytes, we exposed adipocytes to 25 μg/mL HepG2 exosomes for 24 h and evaluated the transcriptomic alterations using gene expression profiling. Seven hundred twenty-five upregulated and 648 downregulated genes were identified (Fig. 2a), and the top 10 up- and downregulated genes in HepG2 exosome-treated adipocytes (exo-adipocytes) compared to control are shown in Fig. 2b. Interestingly, unsupervised clustering identified expression changes in gene signatures related to inflammation in exosome-treated adipocytes (Fig. 2c). Considering that cancer is often associated with an inflammatory milieu, we began to explore whether HepG2 exosomes could change inflammatory response in adipocytes. In our previous report, we found that lung tumor exosomes trigger the release of cytokines including IL-6, IL-8, and MCP-1 in MSC [24]. Therefore, we extended this analysis in adipocytes and confirmed the enhanced release of IL-6, IL-8, and MCP-1 by qRT-PCR (Fig. 2d left) and ELISA (Fig. 2d right). To determine whether increased expression of cytokines in exosome-treated adipocytes was concentration-dependent, we treated adipocytes with different concentrations of HepG2 exosomes for 24 h and found a partial dose-dependent effect of exosomes on the secretion of IL-6, IL-8, and MCP-1 (Fig. 2e).

### exo-adipocytes promote tumor growth in vivo

**Fig. 3:**
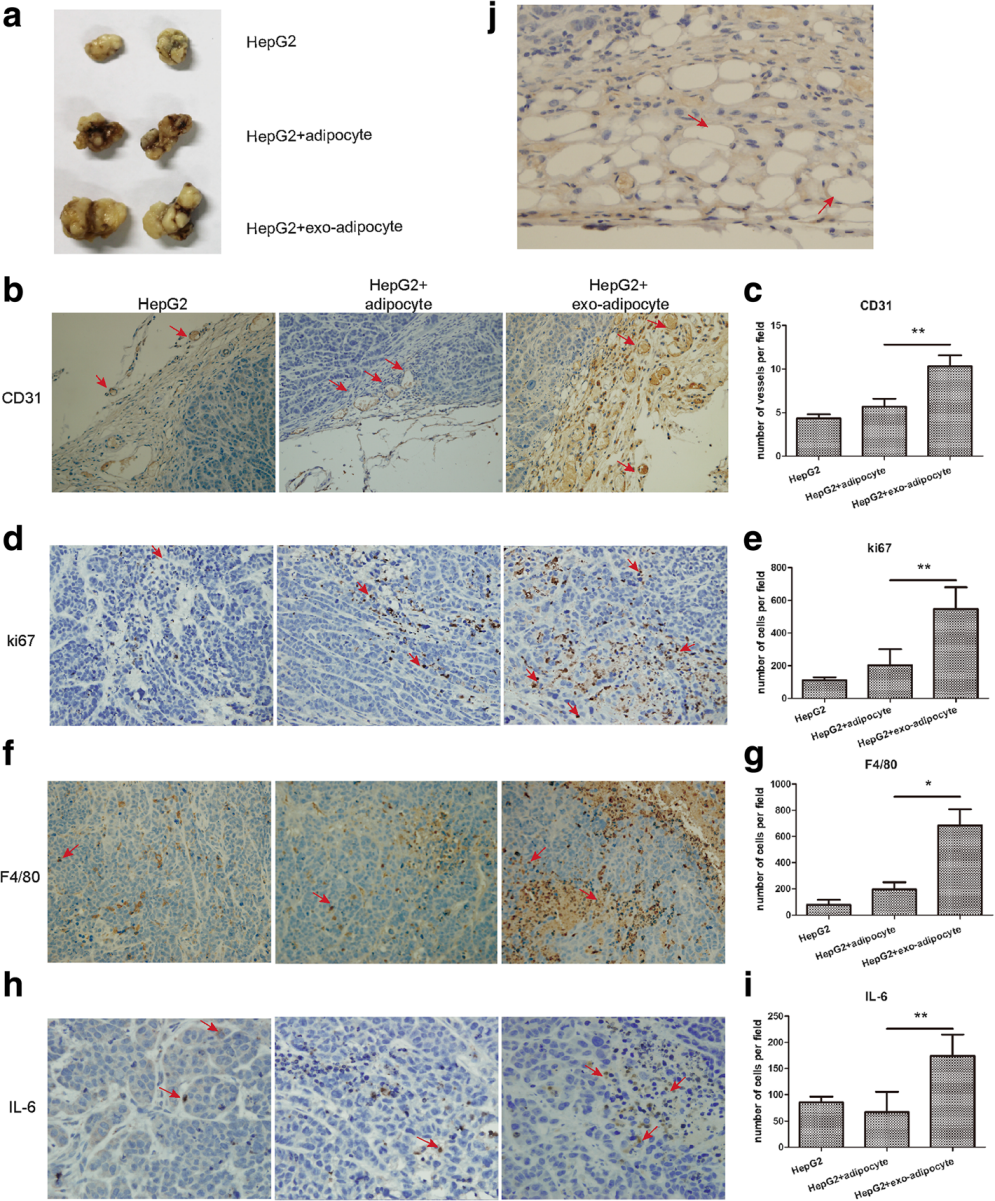
exo-adipocytes promote tumor growth, enhance angiogenesis, and recruited macrophages in vivo. **a** Representative photographs of HepG2 tumors generated from nude mice injected with tumor cells alone or co-injected with exo-adipocytes or control adipocytes at a ratio of 10:1. **b** IHC staining of blood vessel density in tumor sections from xenografts by staining with anti-CD31 antibody. **c** CD31-positive cells were quantified, and the data represents the mean number of CD31 + cells per 200× field (three fields per group). **d** Detection of proliferating cells in tumors with IHC staining using the anti-Ki67 antibody. Representatives of Ki67 staining from each group are shown (magnification × 200). **e** Ki67 staining-positive cells were quantified, and the data represent the mean number per 200× field (three fields per group). **f** The macrophage infiltration was examined by detecting the number of F4/80-positive cells using immunohistochemical staining in the tumor tissues harvested. Representatives of F4/80 staining from each group are shown (magnification × 200). **g** F4/80 + macrophages were quantified, and the data represent the mean number of F4/80+ macrophages per 200× field (three fields per group). **h** Detection of IL-6 expression in tumors. **i** IL-6 expression was quantified, and the data represent the mean number of IL-6+ cells per 200× field (three fields per group). **j** A representative H&E staining of tumor sections demonstrated presence of adipocytes in mice injected with exo-adipocytes or adipocytes

Considering the important role of inflammatory cytokines in tumor development, we next analyzed whether exo-adipocytes affected HepG2 cells and provided a benefit for tumor growth by using a nude mouse xenograft tumor model. HepG2 cells were subcutaneously co-implanted with exo-adipocytes, adipocytes, or PBS at a ratio of 10:1. Coinjection with exo-adipocytes resulted in an increased tumor weight compared with tumor cells injected with adipocytes or PBS (Fig. 3a).To determine the effect of exo-adipocytes on angiogenesis and proliferation of tumor cells in vivo, we performed IHC staining to detect CD31 and Ki67. Coinjected exo-adipocytes could enhance the vascular density as demonstrated by the increased expression of CD31 (Fig. 3b,c). Figure 3d, e revealed that the numbers of Ki67-positive cells were increased significantly in the presence of exo-adipocytes. When the tumors were excised for assessment of immune cell infiltration, we observed that the number of F4/80 macrophages was higher in tumors receiving exo-adipocytes than those receiving control adipocytes (Fig. 3f, g). Previous studies suggest IL-6 to be a major regulator of tumor-stroma interaction in cancer microenvironment [25]. Here, we examined IL-6 expression levels in tumor sections and found increased IL-6 protein levels (Fig. 3h, i), consistent with the upregulation of IL-6 genes shown in Fig. 2e. In addition, we observed the appearance of adipocytes among cancer cells in the tumor sections, suggesting that adipocytes were not consumed by neighboring cancer cells during the 4-week tumorigenesis process (Fig. 3j). Taken together, exo-adipocytes were endowed with a capability by tumor exosomes to promote tumor growth, enhance angiogenesis, and recruit macrophages in vivo.

### exo-adipocyte-conditioned medium is chemotaxic and promotes HepG2 migration

**Fig. 4:**
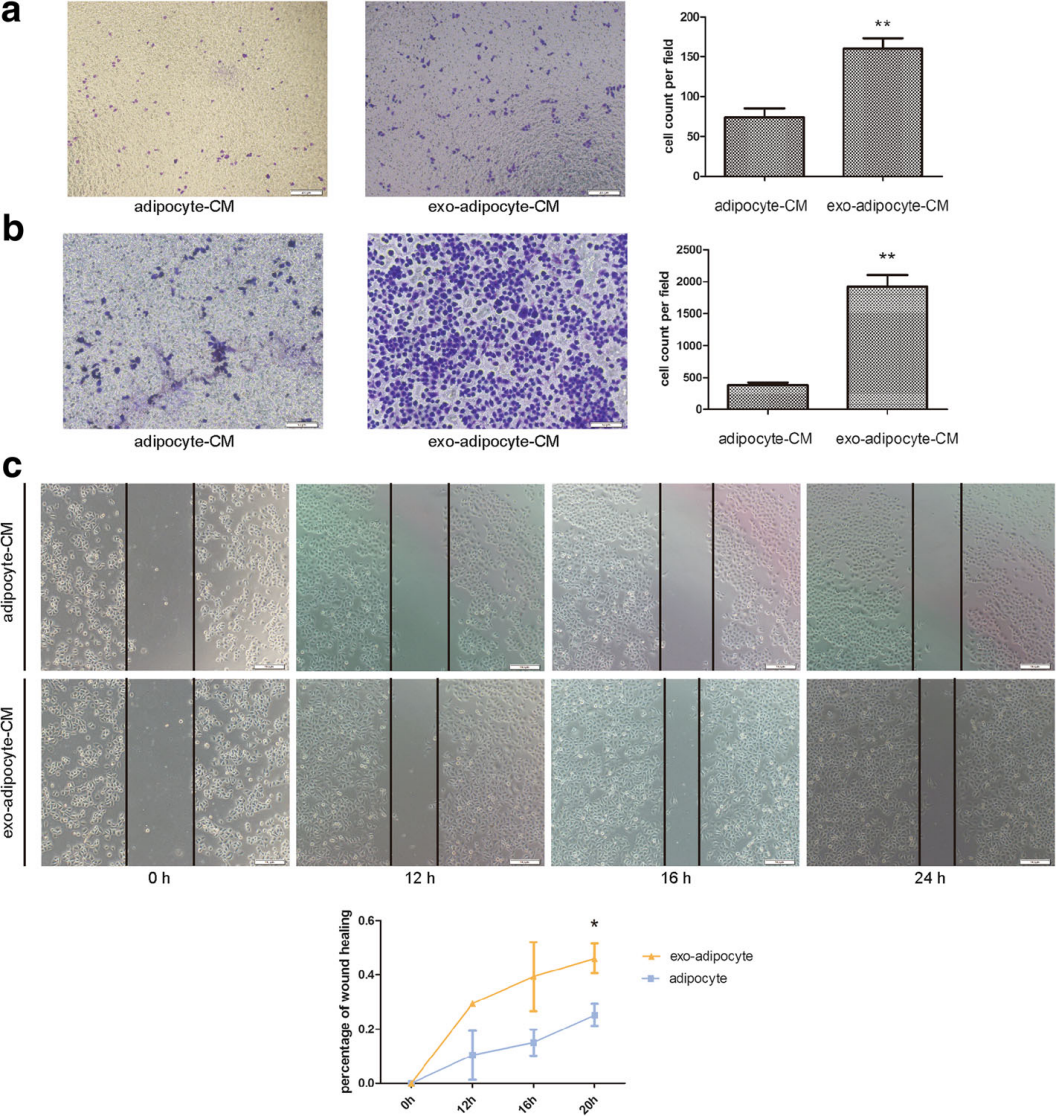
exo-adipocyte-conditioned medium is chemotactic and promotes HepG2 migration. **a** Transwell migration assays showed that THP-1 cells were more chemotactic toward exo-adipocyte-conditioned medium than adipocyte-conditioned medium. Left is a representative microscopic image of crystal violet staining; right shows the statistical results. **b** Transwell migration assays showed that HepG2 cells were more chemotactic toward exo-adipocyte-conditioned medium than adipocyte-conditioned medium. Left is a representative microscopic image of crystal violet staining; right shows the statistical results. **c** Migratory abilities of HepG2 co-cultured with exo-adipocyte-conditioned medium or adipocyte-conditioned medium were determined by wound healing assay

The increased number of F4/80 macrophages in tumors receiving exo-adipocytes prompted us to investigate the effect of exo-adipocyte-conditioned medium on THP-1 cells in vitro as THP-1 cells are one of the most widely used cell lines to investigate the function and regulation of macrophages [26, 27]. We found that exo-adipocyte-conditioned medium was more chemotactic than adipocyte-conditioned medium for THP-1 cells (Fig. 4a). Additionally, a similar effect was observed for HepG2 tumor cells (Fig. 4b). We then examined whether exo-adipocyte-conditioned medium could affect tumor cell migration. As expected, compared with the adipocyte-conditioned medium, exo-adipocyte-conditioned medium increased the migration capacity of HepG2 (Fig. 4c).

### exo-adipocyte-conditioned medium enhanced tube formation of HUVECs

**Fig. 5:**
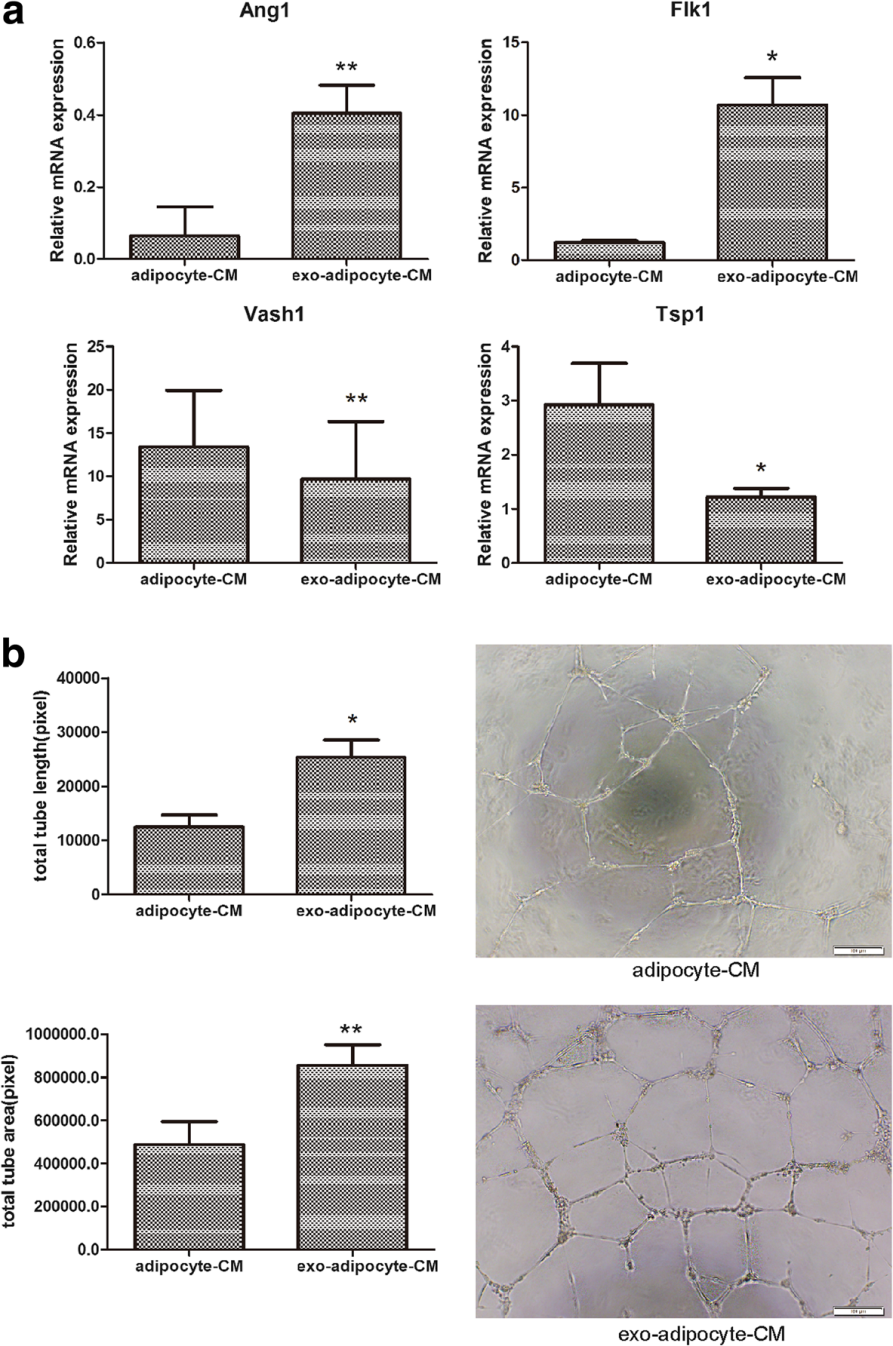
exo-adipocyte-conditioned medium enhances tube formation of HUVECs. **a** HUVECs were incubated with exo-adipocyte-conditioned medium or adipocyte-conditioned medium for 48 h. The mRNA levels of Ang1, Flk1, Vash1, and TSP1 were evaluated by qRT-PCR. Results are mean ± SD (*n* = 3 for each group). **b** Compared with adipocyte-conditioned medium, exo-adipocyte-conditioned medium increased HUVEC tube formation in vitro. Scale bar, 100 μm. Right—representative photograph of tube formation. Left—calculations in three randomly selected fields. Results are mean ± SD (Student’s *t* test)

To confirm our in vivo findings that exo-adipocytes promote angiogenesis in tumors, we examined the effect of exo-adipocyte-conditioned medium on HUVECs. Forty-eight hours after exposure to exo-adipocyte-conditioned medium, HUVECs exhibited upregulated expression of pro-angiogenic genes Ang1 and Flk1 as well as downregulated the expression of anti-angiogenic genes Vash1 and TSP1 (Fig. 5a). We further evaluated the effects of exo-adipocyte-conditioned medium on HUVECs tube formation. As expected, tube formation of HUVECs was significantly increased in the presence of exo-adipocyte-conditioned medium as demonstrated by the increase in tube lengths and areas (Fig. 5b). Collectively, our results suggest that exo-adipocyte-conditioned medium promoted angiogenesis of endothelial cells both in vitro and in vivo.

### HepG2 exosomes activate various kinases and NF-κB signaling pathway in adipocytes

**Fig. 6:**
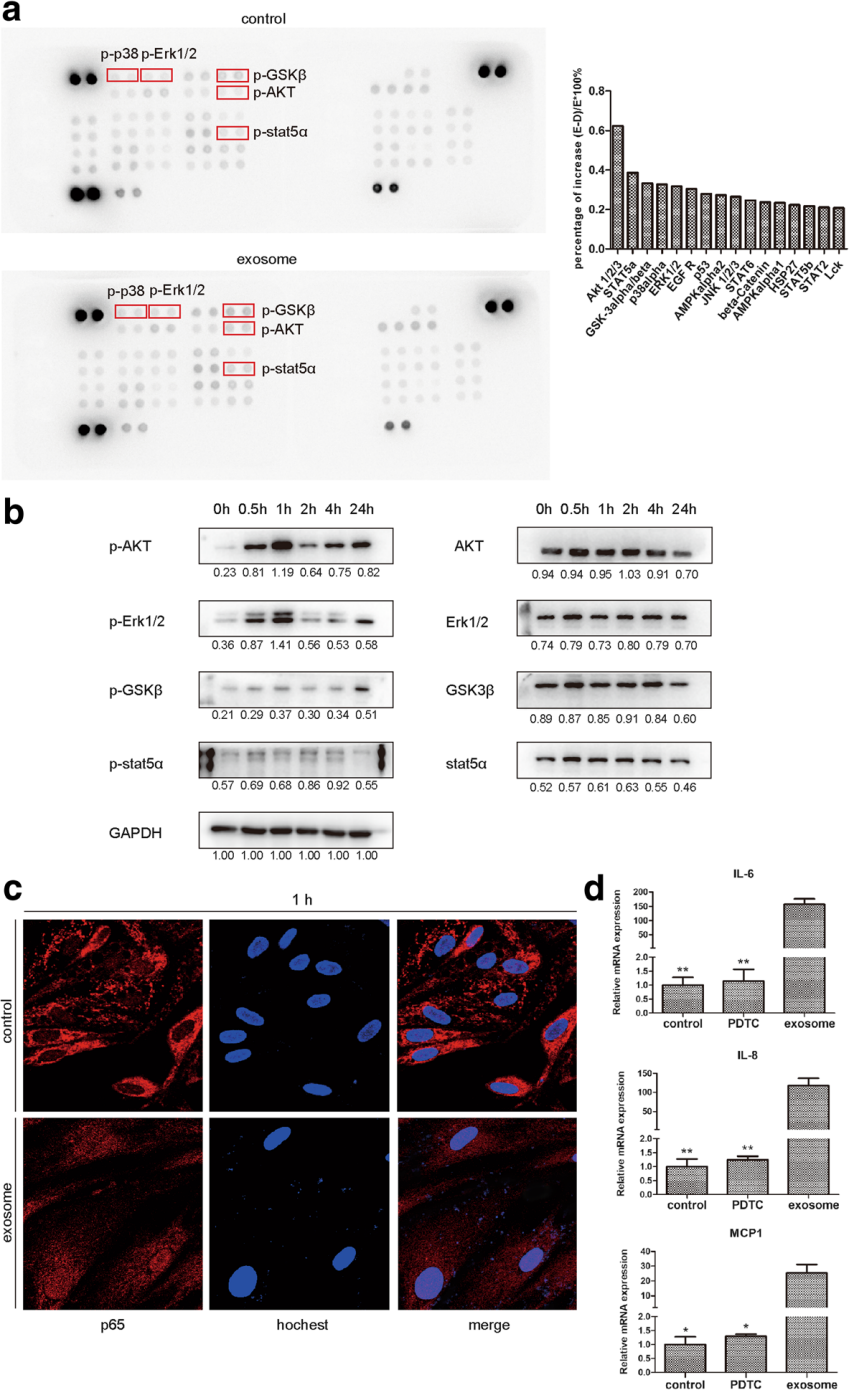
HepG2 exosomes activate several kinases and NF-κB in adipocytes. **a** Phospho-kinase antibody array was performed on protein lysates from adipocytes treated with or without HepG2 exosomes. Data (right) are reported as percentage of increase. The percentage was calculated as (exosome − control)/exosome × 100%, and percentage over 20% is considered statistically significant. The top 5 kinases with an increased phosphorylation were highlighted by red boxes in the left panel. **b** Phosphorylation of AKT, ERK1/2, STAT5α, and GSK3β was confirmed by Western blot. GAPDH was used as loading control. **c** Representative immunofluorescence staining images of nuclear translocation of p65 in HepG2 exosome-treated adipocytes. Red (anti-p65 antibody), blue (Hochest). **d** Relative mRNA expression of IL-6, IL-8, and MCP-1 in adipocytes treated with exosome in the presence or absence of NF-κB inhibitor (*P < 0.05, **P < 0.01)

To identify which signaling pathways were activated by HepG2 exosomes, we performed phospho-kinase antibody array in adipocytes treated with or without HepG2 exosomes for 1 h. As shown in Fig. 6a, of the 43 kinases examined, 15 was detected to have an increase of phosphorylation in exo-adipocytes. The top 5 increased kinases were AKT, STAT5α, GSK3 alpha/beta, p38 alpha, and ERK1/2. Using Western blot, we confirmed the strong and rapid activation of AKT, STAT5α, ERK1/2, and GSK3β (Fig. 6b). Since several kinases activated in adipocytes such as AKT, ERK1/2, and GSK3β are closely associated with NF-κB signaling pathway, we investigated the possible activation of NF-κB after HepG2 exosome treatment. Figure 6c showed the translocation of active p65 from the cytoplasm to the nucleus.

Moreover, when NFκB inhibitor PDTC was added, the enhanced expression of IL-6, IL-8, and MCP-1 induced by HepG2 exosomes in adipocytes was reduced (Fig. 6d). Taken together, these results demonstrated that HepG2 exosomes are able to activate various kinases and NF-κB signaling pathway in adipocytes.

### Proteomic analysis of HepG2 exosomes

**Fig. 7:**
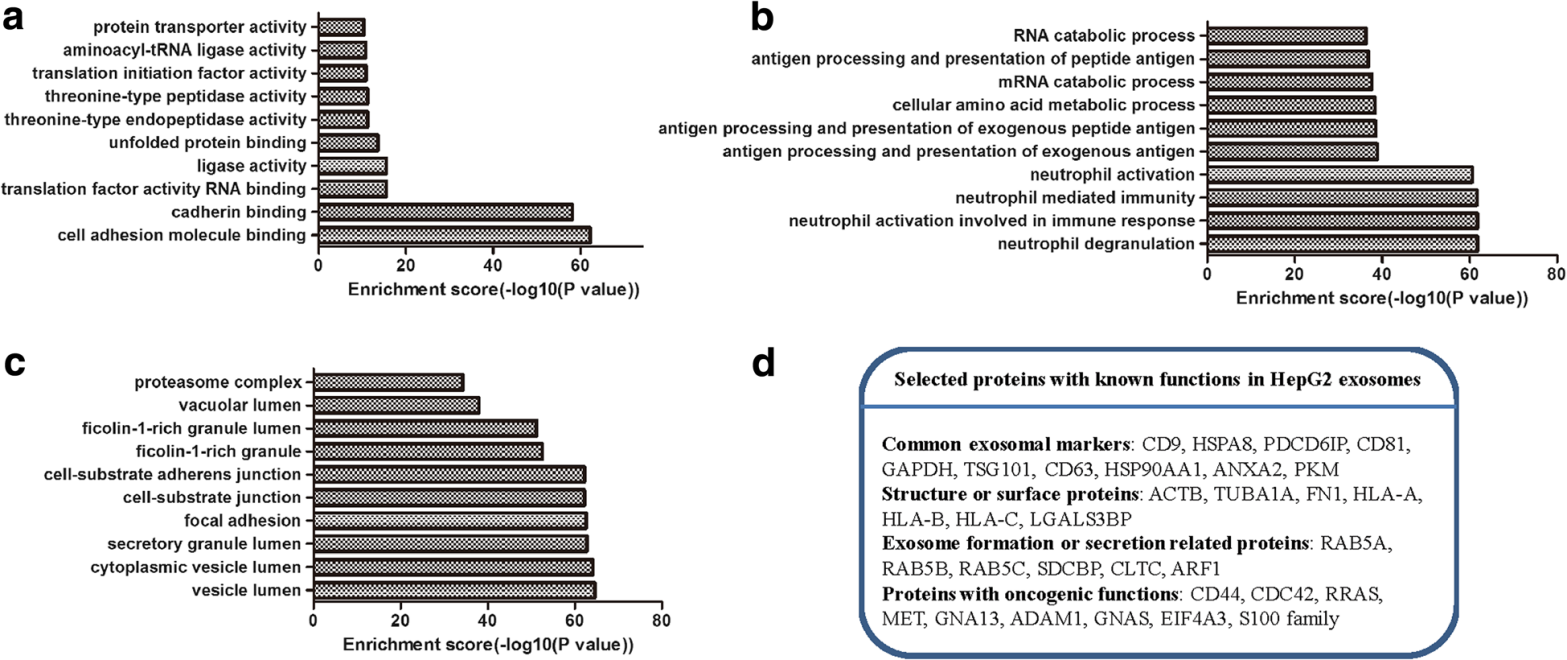
Proteomic analysis of proteins recovered from HepG2 exosomes. The identified proteins were classified according to biological process (**a**), molecular function (**b**), and cellular component (**c**). **d** Selected proteins with known functions in HepG2 exosomes

Finally, we used mass spectrometry to characterize proteins contained within HepG2 exosomes. One thousand four hundred twenty-eight proteins were detected in exosomes, which were classified by GO annotation according to biological process, cellular component, and molecular function. The results showed a high prevalence of proteins involved in immune responses (“biological process,” Fig. 7a), proteins with binding activity (“molecular function,” Fig. 7b), and a high proportion of proteins associated with vesicle and granule (“cellular compartment,” Fig. 7c). We selected 32 proteins with known functions according to Exocarta (http://www.exocarta.org/). As demonstrated in Fig. 7d, these selected proteins included common exosomal markers, structure or surface proteins, exosomal formation or secretion-related proteins, and oncogenic proteins. Future studies are required to identify which proteins are involved in the modification of adipocytes into tumor-promoting cells.

## Discussion

Tumor initiation and progression rely on the dynamic interactions between malignant tumor cells and multiple normal cell types within its microenvironment such as fibroblasts, various immune cells, endothelial cells, and adipocytes. Of these cell types, adipocytes are probably the least well studied, although they represent a significant part of the tissue surrounding a tumor [28]. Emerging evidence suggests that adipocytes should not be considered simply as an energy-storage depot. Instead, adipose tissue can play a central role in both endocrine and metabolic processes by producing a battery of factors including growth factors and adipokines [29]. Thus, understanding how obesity and adipose tissue-related factors are connected to tumor development is paramount. In 2010, Dirat’s group coined the term “cancer-associated adipocytes (CAA)” to demonstrate the bidirectional crosstalk between breast cancer cells and tumor-surrounding adipocytes and that CAA are a key player in tumor progression [30]. Subsequently, several studies also showed the existence of the putative CAA in the vicinity of cancer cells [31, 32]. Here, we chose adipocytes as a cellular model which are differentiated by culturing human MSCs under adipogenic conditions and are fully characterized by morphology, staining, and marker gene expression. We demonstrated that HCC cell line HepG2-derived exosomes could be actively incorporated by adipocytes and convert adipocytes into tumor-promoting cells (exo-adipocytes). In the mouse xenograft model, we found that exo-adipocytes promoted tumor growth and enhanced angiogenesis. Fujisaki et al. reported that in the presence of breast cancer cell lines MCF7 and MDA-MB-231, adipocytes reverted to an immature and proliferative phenotype of CAA that could promote cancer cell migration [33]. Lee et al. found that when indirectly co-cultured with breast cancer cells, adipocytes would be transited into CAA, resulting in proliferation-enhancing effect in ER-positive breast cancer cells such as MCF7 and ZR-75-1 but not in ER-negative cells [34]. Thus, we postulate that the exo-adipocytes in our study are a kind of CAA as they exhibit tumor-promoting capacity and higher expression of pro-inflammatory factors such as IL-6, IL-8, and MCP-1 whose higher expression in CAA has been reported [33, 34]. IL-6 plays diverse regulatory roles in cancer pathogenesis including remodeling the tumor microenvironment, activation of EMT process, and promoting drug resistance [35, 36]. IL-8 is known to be a stimulatory factor for tumor angiogenesis [37], and MCP-1 promotes the recruitment of macrophages into tumors [38]. These cytokines may be at least partially responsible for the tumor-promoting and angiogenesis-enhancing effects of exo-adipocytes.

The regulatory mechanisms of the CAA transition are not clearly understood. In this study, we explored the role of HCC-derived exosomes on the cellular and molecular changes of exo-adipocytes, which further confirmed that tumor cells could use exosomes as a novel way of cell-cell communication. Our study is consistent with previous findings that tumor exosomes from various cancer types can “educate” neighboring cells such as MSCs [39], endothelial cells [40], monocytes [41], and dendritic cells [42]. For example, exosomes from ovarian and breast cancer cells can convert adipose-derived MSCs (AMSC) into myofibroblast-like cells [43, 44] while prostate cancer cell-derived exosomes trigger bone marrow MSCs (BMSC) to differentiate into pro-angiogenic and pro-invasive myofibroblasts [45]. Our results support the postulation that the elements of adipose tissue can also be modified by cancer cells and participate in a highly complex vicious cycle to form a tumor-favorable microenvironment.

How exosomes cause significant cellular and molecular changes in target cells remains an area of intensive research. Using microarray, Fang et al. found that HCC exosomes could deliver miR-1247-3p into fibroblasts and converted them into cancer-associated fibroblast to foster lung metastasis [46]. Using proteomic analysis, He et al. revealed that exosomes derived from metastatic HCC cell lines carried a large number of protumorigenic proteins, such as MET protooncogene, S100 family members, and the caveolins [19]. Here, we also detected common exosomal markers, structure or surface proteins, exosomal formation or secretion-related proteins, and oncogenic proteins in HepG2 exosomes. Upon taking up HCC exosomes, 725 upregulated and 648 downregulated genes were identified, and several cell signaling pathways were activated. In our previous study [24], we found that lung tumor exosomes could activate NFκB signaling pathway through HSP70/TLR2. Here, we also detected the activation of the NFκB signaling pathway. However, several questions remain for future investigation, including which receptors on the surface of adipocytes participated in HCC exosome internalization and how the internalized exosome cargos activated the downstream signaling pathways.

## Conclusions

**Fig. 8:**
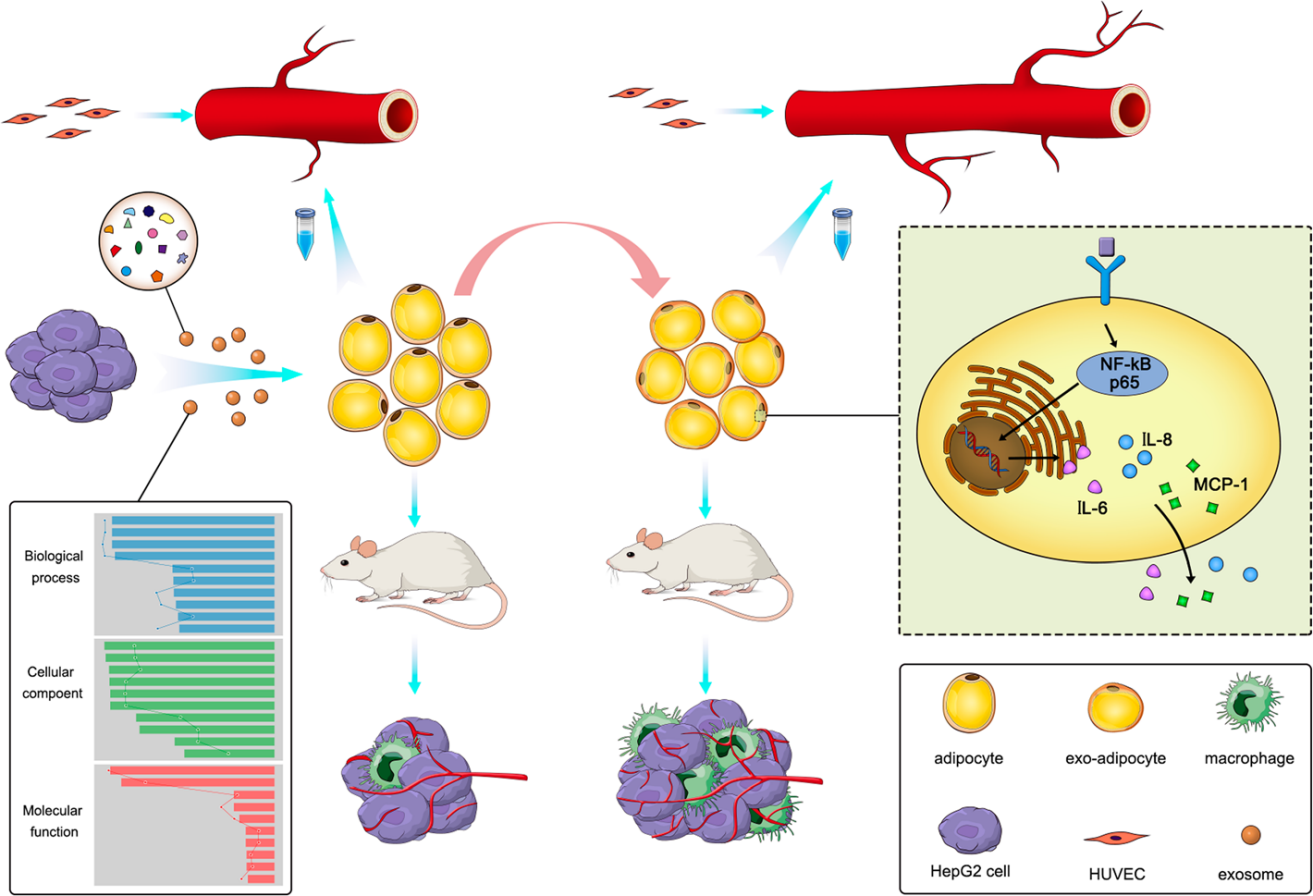
A schematic illustration demonstrates that HCC-derived exosomes can convert adipocytes into tumor-promoting cells that could promote tumor growth, enhance angiogenesis, and recruit macrophages

Collectively, our data indicated that (i) HCC tumor-derived exosomes were actively incorporated into adipocytes and dramatically changed adipocytes transcripome and cytokine secretion; (ii) exo-adipocytes strongly supported tumor growth, enhanced angiogenesis, and recruited more macrophages; and (iii) several kinases and NF-κB signaling pathway were activated in exo-adipocytes (Fig. 8). Our results provide new insights into the concept that tumor cell can educate surrounding adipocytes to create a favorable microenvironment for tumor progression and that this effect might be amplified in overweight patients.

## Abbreviations

EDTA: Ethylenediaminetetraacetic acid
ELISA: Enzyme-linked immunosorbent assay
exo-adipocytes: Exosome-treated adipocytes
FBS: Fetal bovine serum
H-DMEM: High glucose of Dulbecco’s modified Eagle’s medium
HE: Hematoxylin-eosin
MCP-1: Monocyte chemotactic protein 1
MSCs: Mesenchymal stem cells
NF-κB: Nuclear factor kappa-light-chain-enhancer of activated B cells
IL: Interleukin
qRT-PCR: Quantitative real-time PCR
